# Quality of Life after Surgical Treatment for Chronic Otitis Media: A Systematic Review of the Literature

**DOI:** 10.3390/jpm12121959

**Published:** 2022-11-27

**Authors:** Daniela Lucidi, Carla Cantaffa, Riccardo Nocini, Andrea Martone, Matteo Alicandri-Ciufelli, Daniele Marchioni, Livio Presutti, Giulia Molinari

**Affiliations:** 1 Department of Otolaryngology—Head and Neck Surgery, University Hospital of Modena, 41124 Modena, Italy; 2 Unit of Otolaryngology—Head and Neck Department, University of Verona, 37126 Verona, Italy; 3 Otolaryngology and Audiology Unit, IRCCS Azienda Ospedaliero-Universitaria Policlinico di Sant’Orsola, 40138 Bologna, Italy; 4 Department of Specialist, Diagnostic and Experimental Medicine, Alma Mater Studiorum University, 40138 Bologna, Italy

**Keywords:** quality of life, tympanoplasty, chronic otitis media, cholesteatoma, endoscopic ear surgery, canal wall down

## Abstract

This systematic review aims to (a) define what instruments are available to measure quality of life (QoL) in patients undergoing tympanoplasty for chronic otitis media (COM) and what is the most commonly selected timing to do so; (b) compare outcomes from different surgical techniques; and (c) describe any reported correlation between subjective and functional results. This review was conducted following the PRISMA statement recommendations. Of the 151 articles screened, 24 were included. Most studies had a prospective design. The mean age at surgery was 44.5 years. A microscopic retroauricular approach was the most common surgical technique. Most articles included both primary and revision surgeries. The most commonly used questionnaire was the Glasgow Benefit Inventory (GBI), followed by the Chronic Ear Survey (CES), the Chronic Otitis Media Outcome Test 15 (COMOT-15) and the Zurich Chronic Middle Ear Inventory (ZCMEI-21). Questionnaires were administered about 12 months after surgery in most studies. Ten studies reported possible associations between hearing results and QoL. QoL assessment after COM surgery variably relies on disease-specific and non-specific questionnaires. Patients are usually evaluated 12 months after surgery, and this appears to be a suitable timing to contrast the possible bias effect of different tympanoplasty techniques associated with different healing times. A comparison between QoL outcomes in different surgical approaches cannot be made, as several influencing factors have not been detailed in the included studies. Few studies have investigated the correlation between subjective and objective outcomes of tympanoplasty for COM so far.

## 1. Introduction

Chronic otitis media (COM) patients typically suffer from ear discharge, pain and hearing impairment. One or more of these symptoms may persist to some degree even after surgery, sometimes causing patients burdensome restrictions in daily life and social activities (especially water contact) and frequent use of the healthcare system. In recent years, a constellation of studies with focus on quality of life (QoL) has populated the scientific literature on ear surgery. COM makes no exception, as, with its burdening symptoms, it could greatly influence a patient’s QoL. The first study on this topic dates back to 2008 [1], highlighting that the interest in subjective outcomes of ear surgery is somewhat new. So far, in fact, the success of COM treatment has been based exclusively on objective outcomes, such as recurrence rates and hearing function recovery.

Studies regarding QoL after COM surgery are few and heterogeneous, especially regarding the instruments used to evaluate QoL. A number of tools, such as the Glasgow Benefit Inventory (GBI), Short Form-36 (SF-36), Hearing Handicap Inventory for Adults (HHIA) and modified Amsterdam Inventory for Auditory Disability and Handicap Score (mAIAD), which have been employed in QoL studies after ear surgery, have some limitations in their suitability for the assessment of specific otologic conditions because they are either too generic or they evaluate only a subset of COM-related symptoms, most frequently hearing loss [2]. Moreover, different surgical techniques are currently available for COM treatment; among them, the endoscopic technique has been developed as a minimally invasive approach, allowing a favorable intraoperative view, better cosmetic results and reduced hospitalization [3]. However, results regarding patient-reported outcomes after endoscopic treatment for COM are scarce, as are comparative studies between different surgical approaches.

For all these reasons, interpretation of the results regarding the impact of COM surgery on the psycho-social sphere and global QoL is difficult, at present. Therefore, we sought to systematically review the available literature on QoL after surgery for cholesteatomatous and non-cholesteatomatous otitis media, with the following aims: (a) to define what instruments, specific and non-specific, are available to measure QoL in this subset of patients and what is the most commonly selected timing to administer QoL questionnaires; (b) to compare outcomes from different surgical techniques; (c) to describe any reported correlation between subjective and functional results.

## 2. Methods

This systematic review was conducted following the PRISMA statement recommendations (9). The following search string was run on PubMed, Scopus and Medscape Ovid databases: (“quality of life”[MeSH Terms] OR (“quality”[All Fields] AND “life”[All Fields]) OR “quality of life”[All Fields]) AND (“surgery”[MeSH Subheading] OR “surgery”[All Fields] OR “surgical procedures, operative”[MeSH Terms] OR (“surgical”[All Fields] AND “procedures”[All Fields] AND “operative”[All Fields]) OR “operative surgical procedures”[All Fields] OR “general surgery”[MeSH Terms] OR (“general”[All Fields] AND “surgery”[All Fields]) OR “general surgery”[All Fields] OR “surgery s”[All Fields] OR “surgerys”[All Fields] OR “surgeries”[All Fields] OR (“postoperative period”[MeSH Terms] OR (“postoperative”[All Fields] AND “period”[All Fields]) OR “postoperative period”[All Fields] OR “postop”[All Fields] OR “postoperative”[All Fields] OR “postoperatively”[All Fields] OR “postoperatives”[All Fields])) AND (“tympanoplasty”[MeSH Terms] OR “tympanoplasty”[All Fields] OR “tympanoplasties”[All Fields] OR (“cholesteatoma”[MeSH Terms] OR “cholesteatoma”[All Fields] OR “cholesteatomas”[All Fields]) OR “chronic otitis media”[All Fields]).

After running the above search string in January 2022, 151 titles and abstracts were obtained and screened independently by two of the authors (RN, AM), who subsequently met and discussed disagreements on citation inclusion. Inclusion criteria for citations were mention of patients affected by chronic otitis media; mention of patients undergoing ear surgery; English, French, and Spanish language; and availability of the abstract. Exclusion criteria were animal model studies, systematic reviews, response letters and validations studies, and subjects totally unrelated to chronic otitis media.

**Figure 1:**
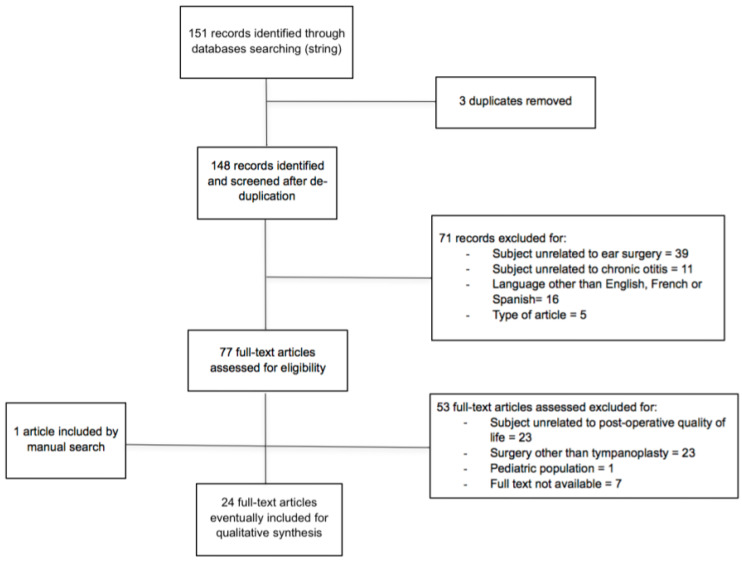
Flow chart for study selection.

Subsequently, the full-text articles identified underwent a second screening by the same two authors. Full-text articles were considered regardless of their study design, in order not to miss any relevant data. Full-texts were included if they concerned patients who underwent surgical treatment for chronic otitis media, and if data regarding post-operative QoL were reported. Studies on stapes surgery or cochlear implant were not considered. Procedures such as Eustachian tube balloon dilation and transtympanic tube positioning were considered only if concomitant to tympanoplasty. Those reporting only on postoperative taste function were excluded, as well as studies on exclusive pediatric populations. A further manual check of the references included in the articles was performed and the final number of articles included in the present review was defined. The flowchart of the selection process is described in Figure 1.

The general characteristics of each study were recorded. Relevant data to answer to the key questions of the systematic review were extracted and recorded on a database. Through a qualitative synthesis of the included studies, results were evaluated and discussed. Considering the small number of cases reported, the review protocol was not registered. Due to the nature of this study, it was granted an exemption by the Institutional Review Board of the University Hospitals of the participating centers. There was no funding source for this study.

## 3. Results

Overall, 24 articles were included, for a total number of 1775 patients analyzed (764 males vs. 832 female, M:F ratio = 1:1.1—data not reported in three articles). According to the data reported in 22 of the studies included, the mean age at surgery was 44.5 (range 26–56).

**Figure 2:**
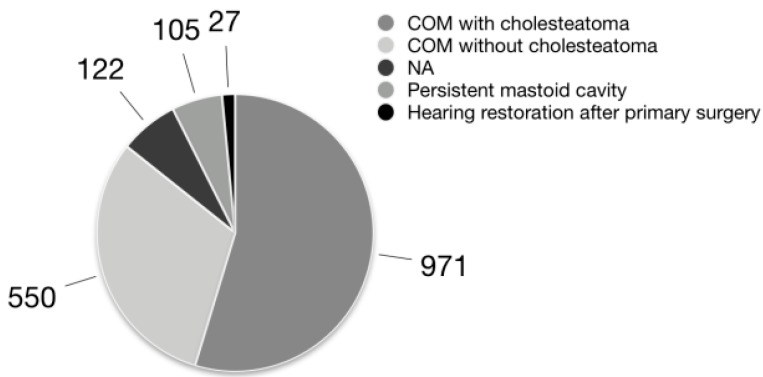
Patient distribution according to the indications for surgery. COM: chronic otitis media; NA: not available.

Table 1 highlights the terminology used to identify COM and its variants, while Figure 2 shows the distribution of the surgical cohorts/patients according to the pathologies treated in each study.

**Figure 3:**
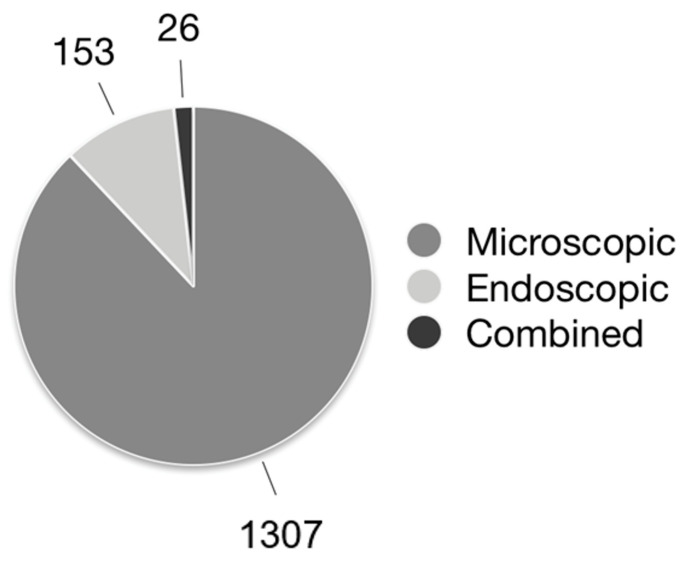
Surgical techniques applied in the included cohorts of patients.

The most commonly applied technique was microscopic retroauricular approach (1307 patients, 73.6%), as shown in Figure 3. Most articles included both primary and revision surgeries (10 papers), while 5 articles focused on primary surgeries and 2 on revision surgeries only. In six articles, the setting of the operations investigated was not specified. Details regarding the surgical approaches are reported in Table 2.

Regarding QoL assessment, all articles reported the use of at least one questionnaire, the most common being the Glasgow Benefit Inventory (GBI), applied in eight studies, followed by the Chronic Ear Survey (CES), the Chronic Otitis Media Outcome Test 15 (COMOT-15) and the Zurich Chronic Middle Ear Inventory (ZCMEI-21), each applied in five studies (Table 3). The timing of questionnaire administration after surgery was also variable, with a mean of 12.75 months (SD 8.12, range 6–28). In two articles, the evaluation of the QoL by questionnaires was integrated with the use of visual analogue scales (VAS) for the rating of QoL-related items, such as ear stuffiness, pain/discomfort in the ear, difficulty in hearing, activity limitation, emotional problems and caregiver’s concern.

Most of the articles did not specify the conditions of the opposite ear in cases of monolateral surgery, and in cases of bilateral surgery (overall 27 patients), data regarding the type of COM (with or without cholesteatoma) were not available. Overall, 837 patients were evaluated for QoL after undergoing primary surgery while 298 were evaluated after revision surgery. 

In sixteen studies, audiometric testing was performed pre- and post-operatively. Of these, ten also reported possible associations between hearing results on audiometric tests and questionnaire outcomes. 

Quality of evidence for each included study was assessed by means of the GRADE system and the risk of bias was graded as either low or high according to Cochrane recommendations, as summarized in Table 2. Most of the studies had a prospective design (16 prospective and 1 prospective randomized). Seven were retrospective, while only one was a double-blind randomized controlled trial. Publication years ranged between 2006 and 2021.

## 4. Discussion

### 4.1. Questionnaires

A number of instruments have been employed as a tool to measure QoL in patients treated for COM, mostly in the form of self-assessment questionnaires. Already existing questionnaires on ear- and auditory-function-related influence on QoL, such as the GBI, HHIA and mAIAD were adapted to patients with this disease. However, they were soon found to be inadequate for the evaluation of the specific impact of COM symptoms on patients’ QoL. The Chronic Ear Survey (CES), introduced in 2000 by Nadol and colleagues at the Massachusetts General Hospital, was the only validated disease-specific questionnaire for COM up to year 2009 [27]. The survey is subdivided into three categories: Activity Restriction, which examines the effect of COM on patient’s daily life; Symptoms; and Medical Resource Utilization, which determines the degree to which antibiotics and health care services are used. This questionnaire, however, has been then deemed incomplete as it does not consider the psychological effects of COM’s symptoms on QoL. In 2009, Baumann and colleagues developed another disease-specific questionnaire, the Chronic Otitis Media Outcome Test 15 (COMOT-15), which includes questions on both the functional sequelae of COM and on the subjective perception of symptoms by affected patients [28]. The questionnaire was subsequently validated on a cohort of 121 patients with chronic suppurative otitis media by the same group in 2011 [4]. The COMOT-15 consists of three subscales called Ear Symptoms, Hearing Function, and Mental Health. In addition, one question on the general evaluation of the impact of COM on QoL and one question regarding the frequency of outpatient visits over a six-month period are included [28].

In 2016, the Zurich Chronic Middle Ear Inventory (ZCMEI-21) was introduced as a disease-specific questionnaire for COM. It consists of four subscales concerning ear signs and symptoms, hearing function, psychosocial impact and the use of medical resources [29].

The CES, COMOT-15 and ZCMEI-21 scores are the only available self-assessment disease-specific questionnaires for COM. Nonetheless, other questionnaires are still found in the literature, making an analytical comparison between different case series hard to perform. For instance, as many as eight of the studies selected for this review applied the nonspecific GBI questionnaire. Those who favor its use claim that, being non-disease specific, this questionnaire has the advantage that it can be employed to compare outcomes of different otorhinolaryngological procedures. It was first developed by Robinson et al. in 1996 [30] and its use in otologic surgery has been validated for middle ear surgery, cochlear implantation and BAHA.

### 4.2. Surgical Technique

Most of the discussion regarding comparison of post-operative QoL according to different tympanoplasty techniques is focused on whether the impact of canal wall down (CWD) tympanoplasty on post-operative QoL is as unbearable as traditionally thought. In fact, it is common knowledge that in the aftermath of a CWD tympanoplasty, patients need to have frequent visits to the outpatient clinic for professional ear cleaning and they have to keep the operated ear away from water. Moreover, they often experience discomfort when using hearing aids and may experience dizziness or vertigo with temperature and pressure changes in the external auditory canal. However, convincing scientific evidence on the matter is currently lacking. In our review, only four studies evaluated QoL after CWD tympanoplasty without mastoid obliteration for COM. One prospective study on 205 patients with and without cholesteatoma demonstrated that the CWD technique was associated with worse scores than CWU tympanoplasty on K-CES (Korean version of the Chronic Ear Survey) on univariate, but not on multivariate analysis [13]. Another study by Lailach and colleagues on 97 patients with cholesteatoma comparing three different surgical techniques, namely CWD tympanoplasty, exclusively transcanal technique (ETC) and combined transcanal transmastoidal technique (TCM), found no statistically significant differences between CWD and closed techniques in overall COMOT-15 scores, even though there was a tendency to a higher frequency of ENT consultations and poorer auditory results for CWD patients [16]. In line with the latter, only the Hearing Function subsection of the COMOT-15 actually differed with statistical significance between patients treated with CWU tympanoplasty and those who underwent CWD tympanoplasty without mastoid obliteration in a comparative study by Lucidi et al. The authors also reported that CES scores were slightly better in the CWU than the CWD group in the Symptoms subsection at 3 months post-operatively, but comparable at the 12 month post-operative assessment, likely due to delayed healing in the CWD group that is intrinsic to the type of surgery. In addition, QoL in patients subjected to CWD was shown to have a significant improvement over time, comparing the 3-month and the 12-month assessment, while in the CWU group only the Activity Restriction subsection of the CES score showed a significant improvement over time [6].

Despite the scarce evidence that CWD is actually associated with a lower QoL with respect to closed techniques, there are a number of studies promoting mastoid obliteration to overcome cavity-related issues. A study comparing 11 patients who underwent secondary mastoid obliteration for cavity-related issues and 11 patients with dry and epithelialized cavities after CWD as control group, the authors observed significantly higher values in overall GBI score and all GBI subscales, except for Social Health, in the study group [12]. Two other studies selected for this review, including one prospective study, demonstrated a significant improvement in QoL after secondary mastoid obliteration in patients with a chronically draining cavity. These studies employed the GBI and ZCMEI-21 scores, respectively [1,20]. On a similar note, no differences in QoL have been reported in CES and COMOT-15 scores comparing CWD with mastoid obliteration with closed techniques [7,16].

Reasonably, these results cannot be discussed without considering the influence of patient selection, as patients who are candidate to open techniques often have disabling symptoms due to extensive disease, whose resolution after surgery likely has a larger impact on QoL than the previously mentioned CWD sequelae. For the same reason, we hypothesize that hearing function is not a main concern for patients that are ultimately treated by CWD, explaining why poorer post-operative auditory function does not necessarily translate in lower overall QoL scores. Supporting this theory, it seems that QoL in patients treated with CWD is less influenced by post-operative AC thresholds with respect to other techniques [16].

Importantly, in comparing QoL in open versus closed techniques, one should also take into consideration the timing of questionnaire administration. There is evidence in the literature that the healing time of mastoidectomy cavities after CWD procedures without mastoid obliteration is longer than in CWD tympanoplasty with obliteration and CWU tympanoplasty [31]. A 12-month follow-up, as employed by most articles included in the study, seems reasonable, as it has been shown that QoL outcomes are not significantly different comparing patients undergoing CWU versus CWD tympanoplasty at this time point. On the contrary, administering questionnaires at an earlier time could be responsible for biased results as patients subjected to open mastoidectomy may have not completely healed yet [6].

Finally, CWD surgery has been demonstrated to be associated with lower cholesteatoma recidivism, which surely has an impact on patients’ QoL, even if not immediately after surgery. Therefore, studies where follow-up is shorter may overlook late recurrences, which do not occur infrequently.

Studies on QoL after endoscopic ear surgery for COM are only a few in the literature, given also the paucity of referral centers where endoscopic ear surgery is routinely performed. We only found two studies assessing the issue, using different questionnaires, but overall demonstrating an improvement in QoL after endoscopic surgery [24,26]. One study attempted to compare the results of endoscopic surgery on QoL with microscopic techniques showing that 68 patients treated with an exclusively endoscopic approach had higher GBI scores in all domains than patients treated with open or combined surgery; however, this difference was not statistically significant. The social domain of the GBI questionnaire was the subscale with the highest improvement in endoscopic cases, tending towards statistical significance in the comparison with the other groups (*p* = 0.52) [24]. In the second study, there was not a control group of patients subjected to open or combined surgery; however, it is interesting to note that the authors have not found any statistically significant difference in post-operative QoL in patients subjected to endoscopic transcanal tympanoplasty versus open centrifugal endoscopic tympanoplasty, probably because in the latter group a limited removal of the posterior external auditory canal wall and no meatoplasty were performed [26].

Results on QoL outcomes after endoscopic surgery outcomes may, however, suffer from a selection bias, as patients who undergo endoscopic tympanoplasty typically have limited disease with a less significant impact on QoL.

### 4.3. Primary vs. Revision Surgery

Revision surgery should be theoretically associated with a worse post-operative QoL with respect to primary surgery, as it is often more extensive. However, studies comparing the subjective outcomes of primary and revision surgery are scarce and sometimes controversial. It seems that total COMOT-15 and CES scores do not differ between the two groups [10,16]. However, Baumann and colleagues observed worse results for the revision surgery group in the Hearing Function subscale of the COMOT-15 [4]. Lailach and colleagues observed no difference in COMOT-15 subscales score between primary and revision surgery patients, but found that improvement in AC threshold from the pre- to post-operative period was significant in the primary surgery group but not in the revision surgery group [16]. On the other hand, in a study by Jung et al., while improvements in total CES score and in the Symptoms subsection were found to be significantly higher in the primary surgery group compared with the revision surgery group, no statistically significant difference was observed in AC threshold and air–bone gap between the two [10]. In summary, it seems that, in revision surgery, QoL does not have a clear association with post-operative hearing function. In support of this hypothesis, Jung and colleagues showed that audiometric test results correlate with CES scores in primary surgery but not in revision surgery patients [10], suggesting that the impact of hearing loss on QoL is somehow dampened in revision patients.

Smaller improvements in QoL from pre- to post-operative assessment in the revision surgery group may be due to patients with recurrent disease being more accustomed to symptoms and tending to complain less, as demonstrated by their higher pre-operative scores in the Symptom section of the CES survey in a study by Jung and colleagues [10]. Additionally, revision surgery is not generally guided by patients symptoms but by clinical discovery of recurrent disease, which generally precedes those. 

Revision surgery patients also seem to have lower scores on the Activity Restriction section of the CES questionnaire post-operatively, reflecting a tendency to be more cautious in their daily life activities [10].

### 4.4. Post-Operative Hearing and QoL

Higher post-operative AC thresholds correlate with worse outcomes on most questionnaires (COMOT-15, CES, ZCMEI-21). Among the reviewed studies, there was only one report of no observed correlation between post-operative hearing status and QoL measured with the GBI questionnaire [14]. The GBI being a-non disease-specific questionnaire, we find it likely that it is not as sensitive as other disease-specific questionnaires in assessing the impact of hearing in tympanoplasty patients. There are discrepancies among studies on which questionnaires subsection is affected the most. Once again, comparisons are made difficult by the use of different surveys and the heterogeneity of selected patients. For instance, as discussed in detail in the previous paragraph, in revision surgery patients the correlation between hearing and QoL is uncertain. In addition, most articles do not comment on the health status of the non-operated ear, which of course has a fundamental impact on overall QoL, especially in regard to hearing abilities.

According to Baumann and colleagues, audiometric test results correlate with two of the three subscales of the COMOT-15, namely Hearing Function and Mental Health [4]. On the other hand, another study by Lucidi et al., observed a correlation between PTA and the Hearing Function subsection alone [6]. Lailach and colleagues report that overall COMOT-15 score and all its subscores significantly correlate to the post-operative hearing level, with the strongest correlation obtained when plotting the postoperative PTA against the Hearing Function subscore [16].

As far as the CES is concerned, Jung and colleagues reported that post-operative AC thresholds had a significant linear correlation with total CES score and the Symptoms subsection [10]. Similarly, Lucidi et al., reported a strong association of post-operative audiometric test results with the Symptoms section of the CES score in a study on QoL after endoscopic tympanoplasty for COM [26]. On the other hand, in a study by Choi and colleagues, hearing results correlated with the Activity Restriction subscale scores, rather than the Symptoms subscale score, in which the questions about hearing loss are included. However, the Activity Restriction subscale contains a question about the restriction of social activity caused by hearing loss, which is what may influence so greatly the questionnaire outcomes in patients with poor post-operative hearing results [30]. COMOT-15 seems to have a higher correlation with the post-operative hearing level, compared to other questionnaires. This may be attributed to a predominant presence of questions on hearing loss and its consequences in the overall score (7 of 13 items) [16].

## 5. Conclusions

COM has an impact on patient’s daily life in more than one aspect, and this accounts for the importance of QoL assessment in the global evaluation of post-operative outcomes. In this context, a variable number of instruments, including disease-specific and nonspecific questionnaires, have been employed. According to our review, patients are usually evaluated 12 months after surgery, and this appears to be a suitable timing to contrast the possible bias effect of different healing times associated with different tympanoplasty techniques. A comparison between QoL outcomes in different surgical approaches cannot be made, as pre-operative patient selection and patient’s expectations have not been detailed in the included studies. Despite the evidence that the impact of hearing function recovery on QoL after surgery seems to be dampened in patients treated with open techniques and in revision surgery patients, few studies have investigated the correlation between subjective and objective outcomes of tympanoplasty for COM so far.

## Figures and Tables

**Table 1:** Variability of the terminology used in the included articles to refer to chronic otitis media with and without cholesteatoma.

Terminology to Identify Chronic Otitis Media and Its Variants as the Treated Pathology	Number of Articles Using It
Chronic otitis media (COM) with or without cholesteatoma	7
Cholesteatoma	6
Chronic suppurative otitis media (CSOM) with or without cholesteatoma	4
Cholesteatoma vs. inflammatory mucosa/process	2
Chronic otitis media (COM) vs. cholesteatoma	2
Chronic otitis media squamous disease with cholesteatoma	1
Mucosal vs. squamous chronic otitis media	1
Adhesive otitis media	1

**Table 2:** Summary of included articles.

	Authors	Year	N Of Patients Eventually Included	Surgery	Comparison of Qol between Groups	Questionnaires	Quality of Evidence	Risk of Bias
1	Baumann I. et al. [4]	2011	90	TPL	Preop vs. postop	COMOT-15; SF-36	Low	Low
2	Si Y. et al. [5]	2018	120	TPL with or without ETBD	No surgery vs. ETBD vs. CT vs. CT + ETBD	THI; COMOT-15; ETS	High	Low
3	Lucidi D. et al. [6]	2019	81	CWU TPL and CWD TPL without obliteration	CWU TPL vs. CWD TPL without obliteration	CES; COMOT-15	Moderate	Low
4	Quaranta N. et al. [7]	2014	100	CWU TPL and CWD TPL with obliteration	CWU TPL vs. CWD TPL with obliteration	CES	Moderate	Low
5	Berling Holm K. et al. [8]	2018	37	Middle ear surgery (not better specified)	Preop vs. postop in cholesteatoma group; cholesteatoma vs. otosclerosis	SF-36	Low	Low
6	Baetens W. et al. [9]	2019	26	CWD TPL with obliteration	Preop vs. postop	COMQ-12	Low	High
7	Jung K.H. et al. [10]	2010	41	CWU TPL and CWD TPL	Primary vs. revision surgery	CES	Low	Low
8	Maile E.J. et al. [11]	2015	161	Middle ear surgery (not better specified)	Preop vs. postop	GBI	Low	Low
9	Uluyol S. et al. [12]	2018	22	CWD TPL and CWD TPL with reconstruction	CWD TPL vs. CWD TPL with reconstruction	GBI	Low	Low
10	Choi S.Y. et al. [13]	2012	156	TPL with or without mastoidectomy	Preop vs. postop	CES	Moderate	High
11	Westerberg J. et al. [14]	2020	34	CWU TPL with obliteration	Preop vs. postop	GBI	Low	High
12	Bhatia K. et al. [15]	2016	37	Type 1 TPL	Preop vs. postop	COM-5	Low	High
13	Lailach S. et al. [16]	2015	97	ETC; TCM; CWD TPL with obliteration	ETC vs. TCM vs. CWD with obliteration	COMOT-15	Moderate	Low
14	Bernardeschi D. et al. [17]	2016	39	CWU TPL and CWD TPL with epitympanic and mastoid obliteration with bioactive glass s53p4	CWD TPL VS. CWU TPL; primary versus revision surgery; cholesteatoma vs. non-cholesteatomatous otitis media	GBI and a surgery-specific questionnaire	Low	High
15	Kurien G. et al. [18]	2013	58	CWD TPL with primary or secondary obliteration	Primary vs. secondary obliteration	GBI	Low	High
16	Dornhoffer J.L. et al. [1]	2008	23	Revision of open cavity with secondary obliteration	Preop vs. postop	GBI with three additional, surgery-specific questions	Low	High
17	Weiss N.M., Bächinger D., Rrahmani A., et al. [19]	2020	54	NA	Comparison between patients with different cholesteatoma stage according to the ChOLE classification	ZCMEI-21	Moderate	High
18	Weiss N.M., Bächinger D., Botzen J., et al. [20]	2020	25	Revision of open cavity with secondary obliteration	Preop vs. postop	ZCMEI-21	Low	High
19	Lailach S. et al. [21]	2021	102	CWU TPL and CWD TPL	Preop vs. postop	COMOT-15, ZCMEI-21, SF-36, PHQ-9	Moderate	High
20	Bächinger D., Großmann W. et al. [22]	2021	108	CWU TPL, CWD TPL, secondary obliteration, hearing restoration surgery	Cholesteatoma vs. COM vs. PMC	ZCMEI-21	Low	High
21	Bächinger D., Mlynski R. et al. [23]	2020	103	CWU TPL, CWD TPL, secondary obliteration, hearing restoration surgery	Cholesteatoma vs. COM vs. PMC	ZCMEI-21	Moderate	High
22	Taneja V. et al. [24]	2020	108	TEES, microscopic TPL, micro-endoscopic TPL	Endoscopic vs. microscopic and combined approaches; cholesteatoma vs. non-cholesteatomatous otitis media	GBI	Moderate	Low
23	Mishra A.K. et al. [25]	2021	68	CWD TPL with obliteration	Bone patè vs. bioactive glass obliteration	GBI	High	Low
24	Lucidi D. et al. [26]	2022	85	TEES and open centrifugal endoscopic tympanoplasty	Cholesteatoma vs. COM; primary vs. revision surgery; endoscopic transcanal vs. open centrifugal TPL	CES; DASS-21	Moderate	High

TPL: tympanoplasty; COMOT-15: Chronic Otitis Media Outcome Test 15; SF-36: Short Form 36; ETBD: Eustachian tube balloon dilation; CT: cartilage tympanoplasty; THI: Tinnitus Handicap Inventory; ETS: Eustachian Tube Score; CWU: canal wall up; CWD: canal wall down; CES: Chronic Ear Survey; COMQ-12: Chronic Otitis Media Questionnaire 12; GBI: Glasgow Benefit Inventory; COM-5: Chronic Otitis Media 5; ECT: exclusively transcanal technique; TCM: combined transcanal transmastoidal technique; ZCMEI-21: Zurich Chronic Middle Ear Inventory 21; PHQ-9: Patient Health Questionnaire 9; COM: chronic otitis media; PMC: persistent mastoid cavity; TEES: transcanal endoscopic part surgery; Depression Anxiety Stress Scale 21.

**Table 3:** Subjective questionnaires used to evaluate quality of life in patients undergoing surgery for chronic otitis media.

Questionnaire for Quality of Life Assessment	Number of Articles Using It
GBI	8
CES	5
COMOT-15	5
ZCMEI-21	5
SF-36	3
COM-5	1
SURGERY-SPECIFIC QUESTIONNAIRES	2
COMQ-12	1
PHQ-9	1
THI	1
ETS	1
DASS-21	1

GBI: Glasgow Benefit Inventory; CES: Chronic Ear Survey; COMOT-15: Chronic Otitis Media Outcome Test 15; ZCMEI-21: Zurich Chronic Middle Ear Inventory 21; SF-36: Short Form 36; COM-5: Chronic Otitis Media 5; COMQ-12: Chronic Otitis Media Questionnaire 12; PHQ-9: Patient Health Questionnaire 9; THI: Tinnitus Handicap Inventory; ETS: Eustachian Tube Score; DASS- 21: Depression Anxiety Stress Scale 21.

## Data Availability

The data presented in this study are openly available in online platforms (Pubmed, Scopus and Medscape Ovid).
